# Is levator ani avulsion a risk factor for prolapse recurrence? A systematic review and meta-analysis

**DOI:** 10.1007/s00192-022-05217-2

**Published:** 2022-05-10

**Authors:** Ellen Yeung, Eva Malacova, Christopher Maher

**Affiliations:** 1 Department of Urogynaecology, Women’s and Newborn Services, Royal Brisbane and Women’s Hospital, Brisbane, Australia; 2 The University of Queensland, Saint Lucia, QLD Australia; 3 Statistics Unit, QIMR Berghofer Medical Research Institute, Brisbane, Australia

**Keywords:** Pelvic organ prolapse, Recurrence, Levator ani muscle avulsion, Surgery, Risk factors

## Abstract

**Introduction and hypothesis:**

Levator ani muscle avulsion as a risk factor for prolapse recurrence is not well established. This systematic review was aimed at evaluating the correlation between levator ani avulsion and postoperative prolapse recurrence with meta-analysis, specifically, the risk of subjective or objective prolapse recurrence and reoperation.

**Methods:**

The protocol was registered in the International Prospective Register of Systematic Reviews (registration number CRD42021256675). A systematic literature search was conducted using PubMed, EMBASE and Cochrane Database of Systematic Reviews to identify all peer-reviewed studies that described levator avulsion in women and investigated operative and postoperative outcomes. All peer-reviewed, English-language cohort studies in those with and without levator avulsion with a minimum of 3 months’ follow-up were included. Pooled unadjusted and adjusted odds ratios were calculated for subjective recurrence, objective recurrence and rates of re-operation. The Cochrane Collaboration Risk of Bias In Non-Randomized Studies (RoBINS) and The Grading of Recommendations Assessment, Development and Evaluation (GRADE) tools were used to assess the quality of the studies included.

**Results:**

Twelve studies with a total of 2,637 subjects and a follow-up period 0.3–6.4 years were identified. There were insufficient data to report a pooled adjusted risk for subjective recurrence and reoperation. On low to moderate quality-adjusted data, the pooled odds of objective recurrence was not significantly associated with levator ani avulsion (aOR 1.68; 95% CI 0.78–3.66).

**Conclusion:**

Levator ani avulsion has not been confirmed as a risk factor for objective prolapse recurrence. Further evidence is needed to investigate the correlation between levator ani avulsion and the risk of subjective recurrence and reoperation.

**Supplementary information:**

The online version contains supplementary material available at 10.1007/s00192-022-05217-2

## Introduction

Pelvic organ prolapse is a common condition affecting up to 50% of parous women [1] and approximately 10–20% of women require surgical treatment in their lifetime [2, 3].

Surgical treatment for prolapse has traditionally reported recurrence rates of approximately 10% at 12 months, but some prospective studies have reported this recurrence rate to be over 50% for native tissue prolapse repairs when blinded assessors were employed to assess objective prolapse recurrence on examination [1]. Risk factors for prolapse recurrence include family history of prolapse, previous prolapse recurrence and high preoperative prolapse stage [4–6]. Native tissue vaginal prolapse repair surgery has also previously been shown in large systematic reviews to be associated with increased prolapse awareness, prolapse recurrence and repeat surgery [7, 8].

Levator avulsion is a term used to describe the detachment of the puborectalis muscle from the pelvic side wall at the point of insertion [9]. This can happen in part (“partial avulsion”) or in full (“complete avulsion”), unilaterally or bilaterally. This injury is secondary to vaginal delivery and has been shown to be increased when instrumental assistance is required. It has also been shown to be associated with an increased incidence of prolapse [10]. However, data relating to levator ani avulsion being a risk factor for prolapse recurrence are inconsistent with some demonstrating levator ani muscle avulsion (LAMA) to be a significant risk factor [4, 5], whereas more recent trials did not [11, 12].

Apart from the discordance in the literature regarding levator ani avulsion as a risk factor for prolapse recurrence, many previous studies failed to adjust aforementioned known risk factors of recurrent prolapse in their analyses. They were also largely limited to retrospective studies, did not report on meaningful data such as reoperation rates, and included vaginal mesh surgery, which has now been discontinued in many centres.

Therefore, we aimed to conduct a more contemporary systematic review with multivariate analysis to explore the risk of prolapse recurrence in women with LAMA across all modes of surgical management.

This systematic review of literature and meta-analysis is aimed at evaluating whether LAMA is a potential risk factor for prolapse recurrence and reoperation and at summarising current existing evidence. It will be constructed such that all types of prolapse surgery are included. Subgroup analysis will be undertaken to differentiate outcomes between different surgical routes and types, including the categories of abdominal, vaginal, obliterative, native tissue and mesh. A broader analysis of adjusted values will also be undertaken to account for all known confounding risk factors for prolapse recurrence.

## Materials and methods

This study was conducted as per the Preferred Reporting Items for Systematic Reviews and Meta-Analyses (PRISMA) guidelines [13] and reported as per the Meta-analysis of Observational Studies in Epidemiology (MOOSE) guidelines [14]. The protocol was registered in the PROSPERO Database (registration number CRD42021256675).

### Information sources and search strategy

We conducted a systematic literature review of PubMed, Embase and Cochrane each from inception to 18 June 2021. Our search strategy combined the key words “levator avulsion” AND “surgery”; “levator” AND “prolapse recurrence” AND “surgery” and “risk factors” AND “prolapse recurrence” AND “levator ani” OR “puborectalis”. The complete search strategy and screenshots of each search is shown in Supplementary Table 1.

### Eligibility criteria

Studies were eligible for inclusion if they were peer-reviewed, English-language articles, described a minimum 3-month follow-up, had a control group of women without LAMA and included raw data or odds ratios. Studies were excluded if they did not measure levator ani avulsion, were not published as original articles or were systematic reviews.

For all articles meeting the inclusion criteria and those for which inclusion criteria could not be determined from the abstract, the full text was reviewed. Further exclusion of full-text articles was conducted with the above criteria. Any articles that had been retracted or a subject of concern was not included in our study.

### Study selection and data extraction

Eligible studies were identified by two authors (EY, CM) using the search strategy above. Each abstract was manually classified for inclusion. One author (EY) examined each included paper to extract data regarding type and prevalence of levator avulsion, type of surgery, frequency of prolapse recurrence in women with and without levator injury, requirement for re-operation and other risk factors for prolapse recurrence. Study characteristics that were collected included author, year of publication, type of study and inclusion and exclusion criteria, and length of follow-up. Data were checked independently by another author (CM).

We extracted raw data and odds ratios (ORs) as well as adjusted ORs (aORs) whenever available. We also collated all the variables of papers with adjusted ORs. All measures of effect are reported as ORs with 95% confidence intervals (CIs). Other raw data available in studies but not reported as an OR were converted to ORs.

### Outcomes

The primary outcomes for the assessment of pelvic organ prolapse surgery have previously been defined in the IUGA/ICS Joint Report on terminology for reporting outcomes of surgical procedures for pelvic organ prolapse [15]. This includes the reporting of subjective patient outcomes, objective outcomes on examination and reoperation for recurrent prolapse. These have been demonstrated to be relevant to both the clinician and the patient. Hence, we have used these three criteria as our three primary outcomes.

Subjective outcome was defined as a positive response to patient awareness of a vaginal bulge. Also, those with a positive response to question 3 on the Pelvic Floor Distress Inventory 20 (PFDI-20) (“Do you usually have a bulge or something falling out that you can see or feel in your vaginal area?”)[16] or question 28 on the Australian Pelvic Floor Questionnaire (APFQ) (“Do you have a sensation of tissue protrusion/lump/bulging in your vagina?”) [17] or on any other validated pelvic floor questionnaires.

Objective assessment required a clinical finding of prolapse recorded on multisite vaginal assessment or staging using recognised scoring systems such as the Pelvic Organ Prolapse Quantification (POP-Q) or Baden–Walker Halfway Scoring system. Those with the distal-most portion of the prolapse situated 1 cm above the hymen or lower (ICS/POP-Q ≥ stage 2) are defined as an objective recurrence. Some authors have utilised ultrasound findings to define prolapse recurrence and this has been defined as the finding of the bladder edge ≥10 mm below the symphysis pubis on maximum Valsalva [18]. Recurrent prolapse requiring retreatment with a pessary or reoperation was included as the third primary outcome in this analysis.

Secondary outcomes to be evaluated in separate analyses include whether LAMA is a risk factor for prolapse recurrence in specific surgeries (i.e. native tissue, vaginal mesh, abdominal prolapse surgeries and obliterative procedures) or single-site prolapse recurrence.

### Assessment of risk of bias

The Cochrane Collaboration Risk of Bias In Non-Randomized Studies – of Intervention (ROBINS-I) tool [19] was used to assess the risk of bias due to confounding, selection of participants in the study, classification of interventions, deviations from intended interventions, missing data, measurement of outcomes and selection of reported results. This information was presented using the robvis visualisation tool [20] and classified as low, moderate, serious or critical risk. If there was insufficient or no information to enable a score for bias, this was classified as no information. ClinicalTrials.gov was searched for each study to allow detection of reporting bias.

### Data synthesis

Data were collected for each measurement of recurrence (subjective, objective, reoperation rate) as ORs with 95% CIs. These ORs were then pooled for analysis. Pre-specified subgroup analysis was conducted for each operation type and for single-compartment recurrences.

We estimated pooled ORs by applying a random effects meta-analysis using the inverse variance method. Pooled ORs from all studies were calculated as unadjusted. For all studies that included adjusted logistics regressions in their analysis, we pooled the adjusted ORs.

Statistical heterogeneity of the study results was evaluated using the *I*^*2*^ statistic, which indicates percentage of total variation across all the studies that can be explained by variation rather than chance [21]. Publication bias was investigated using Egger’s test for asymmetry.

All analyses were performed in STATA/IC version 15.0 (StataCorp, College Station, TX, USA).

### Assessment of certainty

The quality of evidence was assessed using the Grading of Recommendations Assessment, Development and Evaluation (GRADE) approach and tabulated using the GRADEpro Guideline Development Tool (GDT) [22].

## Results

### Study selection

Out of an initial 546 articles identified in the search, 288 articles were screened for the inclusion criteria after duplicates, non-English or non-peer-reviewed articles were removed. A total of 29 articles were assessed in their entirety, and out of those, 12 articles were included in the meta-analysis. After exclusion of systematic reviews, individual studies were excluded after full-text review owing to non-routine methods of scoring levator ani avulsion [23, 24], short follow-up period of 6 weeks [25], a lack of a control group [26] and the inclusion of the entire cohort [27] in a subsequent larger study involved in this meta-analysis [28].

**Fig. 1:**
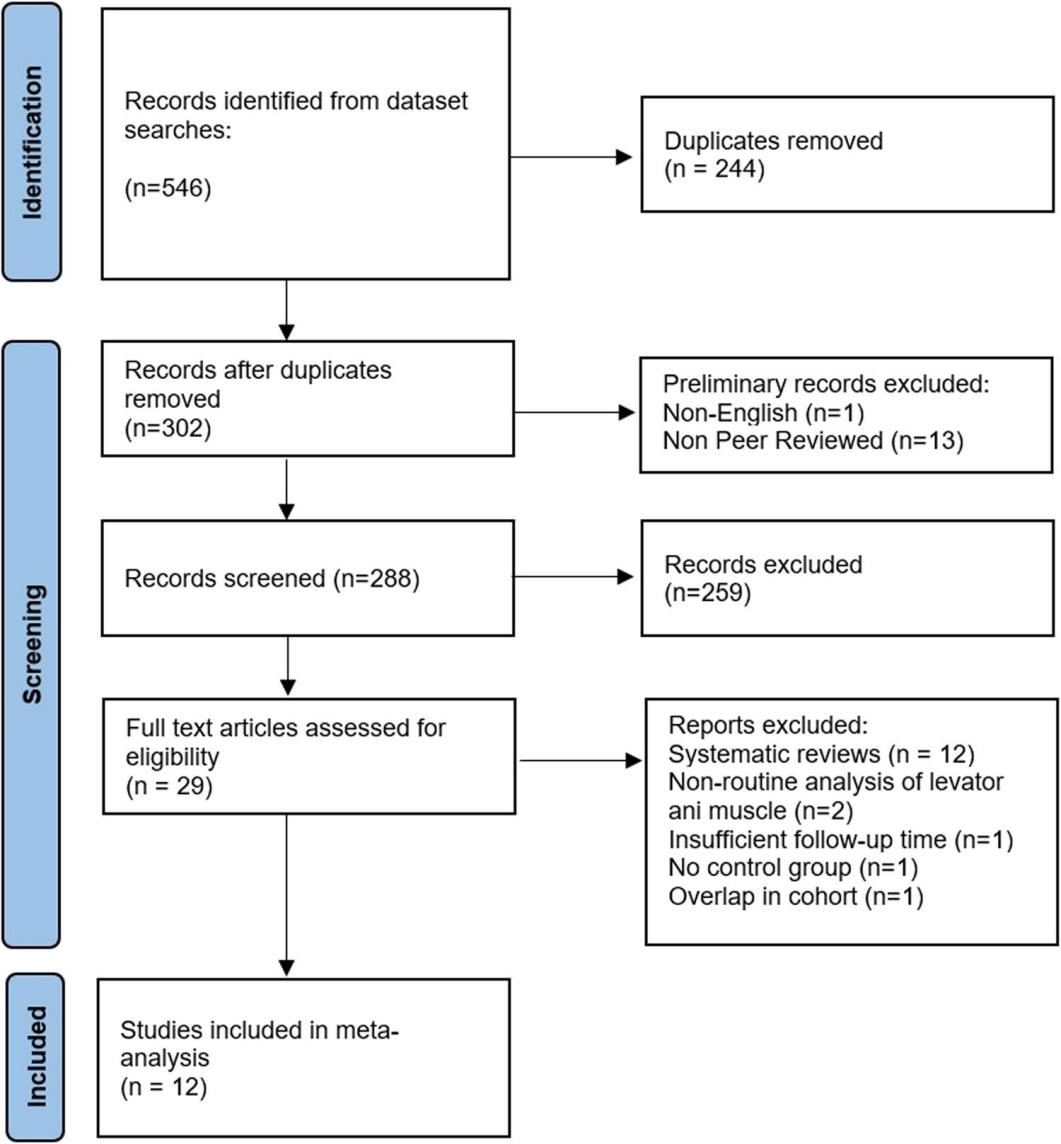
Systematic review of the literature evaluating levator ani avulsion as a potential risk factor for prolapse recurrence. Flow chart summarising study selection and exclusion process

The flowchart summarizing this process and reason for exclusion is presented in Fig. 1.

### Study characteristics

The detailed characteristics of each of the studies included are presented in Table 1. Of the 12 articles included, 3 were prospective [31, 33, 38] and 9 retrospective studies [12, 28–30, 32, 34–37]. A total of 2,637 subjects were analysed, of which at least 1,055 had a diagnosis of either unilateral or bilateral levator ani avulsion. The prevalence of levator avulsion injury in one paper [32] could not be determined as it only reported the ORs for prolapse recurrence after levator ani avulsion injury. This study also analysed patients who had had a hysterectomy for indications other than prolapse and included continence surgery. Hence, only a subgroup of patients from this paper reporting outcomes post-anterior colporrhaphy was included in this meta-analysis. The age of subjects ranged from 58 to 66.9 years and were followed up on average for 2.1 years (range: 0.3-6.4 years).

**Table 1:** Study characteristics, surgeries performed, definition of recurrent prolapse and prevalence of levator ani muscle avulsion (LAMA)

Reference; *N*; mean age	Type of study	LAMA	Mean follow-up (range)	Main surgery	Concomitant surgery	Definition of recurrence
Abdul Jalil et al. [29]; 207; 59 years	Retrospective	111/207 preop, 109/207 postop	1.3 (0.3–5.5) years	One or combination of AC, PC, VH, SSF ± mesh	MUS	Insufficient information
Dietz et al. [30]; 83; 61 years	Retrospective	29/83	4.5 (3–6.4) years	AC	PC, VH, MUS	(a) Symptoms of prolapse OR (b) significant cystocele ICS POP-Q Ba > 0 OR (c) significant cystocele (Ba > 0 on USS (bladder edge >10mm below symphysis pubis on maximum Valsalva
Diez-Itza et al. [31]; 455; 63 years	Prospective	186/439	1 year	AC	PC, VH, MUS	(a) Anterior anatomic recurrence POP-Q > stage 2 (Ba > −1), (b) symptomatic recurrence (yes to question 3 of PFDI-20 questionnaire)
Model et al. [32]; 106	Retrospective	^a^	^a^	AC	^a^	(a) ICS POP-Q > stage 2, (b) patient-reported symptoms of prolapse (vaginal lump or dragging sensation)
Oversand et al. [33]; 189; 61 years	Prospective	96/189	12 (8–21) months	Man	PC	Predefined ‘optimal’ outcome, (a) anterior compartment Ba > −1, (b) mid compartment C > −5, (c) retreatment required within the first year of follow-up (surgery, physiotherapy, pessary); secondary outcome (a) patient0reported subjective satisfaction: cured, improved, unchanged, worsened
Rodrigo et al. [34]; 334; 64 years	Retrospective	130/334	2.51 (0.26–6.39) years	AC ± mesh	PC ± mesh, VH, SSF, MUS	(a) Recurrent symptoms (lump/dragging sensation), (b) ICS POP-Q Ba > −1, (c) cystocele reaching 10 mm below symphysis pubis on Valsalva on translabial USS
Santis-Moya et al. [12]; 134; 60 years	Retrospective	43/134	16 months	SCP	AC, PC, MUS, SH	(a) Anatomical recurrence of any compartment > stage 2 POP-Q and/or retreatment for POP, (b) symptomatic recurrence – affirmative answer to question 3 PFDI-20 questionnaire
Shek et al. [35]; 296; 65 years	Retrospective	117/296	1.8 (0.3–5.6) years	AC + mesh	VH, SSF	(a) Cystocele recurrence ICS POP-Q > Stage 2 OR (b) bladder descent > 10 mm below symphysis pubis on ultrasound
Vergeldt et al. [28]; 287; 58 years	Retrospective	104/279	1–2 years	AC	PC, VH, SSF, man	(a) Anterior anatomical recurrence > stage 2 POP-Q
Wong et al. [36]; 209; 65 years	Retrospective	80/209	2.2 (3 months – 5.6 years) years	AC + mesh	PC ± mesh, VH, SSF, MUS	(a) Subjective prolapse recurrence (symptomatic vaginal lump/bulge/dragging sensation), (b) objective recurrence – clinical cystocele > stage 2 ICS POP-Q or sonographic recurrence (bladder descent > 10 mm below symphysis pubis on USS)
Wong et al. [37]; 183; 63 years	Retrospective	64/183	4.47 years (without mesh), 3.45 years (mesh)	AC ± mesh	PC ± mesh, VH, SSF, MUS	(a) Subjective prolapse recurrence (symptomatic vaginal lump/bulge/dragging sensation), (b) objective recurrence – clinical cystocele > stage 2 ICS POP-Q or sonographic recurrence (bladder descent > 10 mm below symphysis pubis on USS)
Wong et al. [38]; 154; 66.9 years	Prospective	95/154	27.5 months (5 years mesh, 2 years no mesh)	VH + SSF ± AC, PC ± mesh, OR, SCP	MUS	(a) Subjective recurrence: any symptoms of vaginal bulge/dragging sensation, (b) objective recurrence POP-Q > stage 2

*AC* anterior colporrhaphy, *PC* posterior colporrhaphy, *VH* vaginal hysterectomy, *SSF* sacrospinous fixation, *MUS* midurethral sling, *SCP* laparoscopic sacrocolpopexy, *Man* Manchester repair, *SH* subtotal hysterectomy

^a^Could not be ascertained from the paper

Eight studies reported outcomes after anterior repair, of which 4 used native tissue, 2 used anterior mesh repairs and a further 2 studies had a combination of both mesh and non-mesh approaches. Of the remaining 4 studies, 1 analysed outcomes post-Manchester repair [33], another 1 analysed laparoscopic sacrocolpopexy [12], and the last 2 compared a variety of prolapse surgeries [29, 38].

Concomitant surgery was conducted in all studies and included other compartment prolapse repairs, hysterectomy and anti-incontinence procedures.

Of the studies included, 4 papers came from the same centres [34–37] and described the same surgeries undertaken during the reported time periods. The extent of overlap between these studies could not be clarified from the literature.

Of the 12 papers analysed, most of the studies were conducted primarily to define the risk of prolapse recurrence and the effect of a diagnosis of levator avulsion. One paper was aimed at developing a prediction model for cystocele recurrence [28] and another had a primary aim of comparing the agreement between pre- and postoperative ultrasound diagnosis of levator avulsion with a secondary aim of investigating associations between levator avulsion and prolapse recurrence [29]. The preoperative group from this paper was included as part of the cohort for the purposes of this meta-analysis.

### Definitions of recurrence

Most studies analysed subjects for both subjective and objective recurrence of prolapse. Subjective recurrence was either measured as patient-reported symptoms of a bulge or a dragging sensation or with a questionnaire (validated/unvalidated). There was a moderate amount of variation in definitions of objective recurrence. Only 3 studies described objective prolapse recurrence in all compartments [12, 32, 38]. Seven studies described recurrence limited to the anterior compartment [28, 30, 31, 34–37]. One study described a “composite optimal outcome” as a determinant of success [33], whereby an “optimal outcome” was a predefined measurement in both the anterior and the mid compartment. One study [29] did not describe the criteria for prolapse recurrence and was included in the category “objective any compartment” for the purposes of analysis.

For the 12 studies reporting anatomical recurrence, most defined this as an objective clinical finding on examination of a prolapse that was at ICS POP-Q stage 2 or greater. There were five studies that defined objective recurrence as either that diagnosed by examination or that diagnosed using ultrasound.

### Diagnosis of levator ani muscle avulsion

All studies diagnosed LAMA using 4D translabial ultrasound in the method described by Dietz et al. [39]. In 4 studies, LAMA was diagnosed preoperatively [12, 31, 33, 38] and in 6 studies [30, 32, 34–37], LAMA was diagnosed postoperatively. In one study, LAMA was diagnosed pre- and postoperatively [29] and in another, ultrasound assessment was performed either pre- or postoperatively [28]. Where there were two results for levator avulsion measured pre- and postoperatively, the preoperative measurement was taken to reduce risk of bias.

### Prolapse recurrence: subjective

**Fig. 2:**
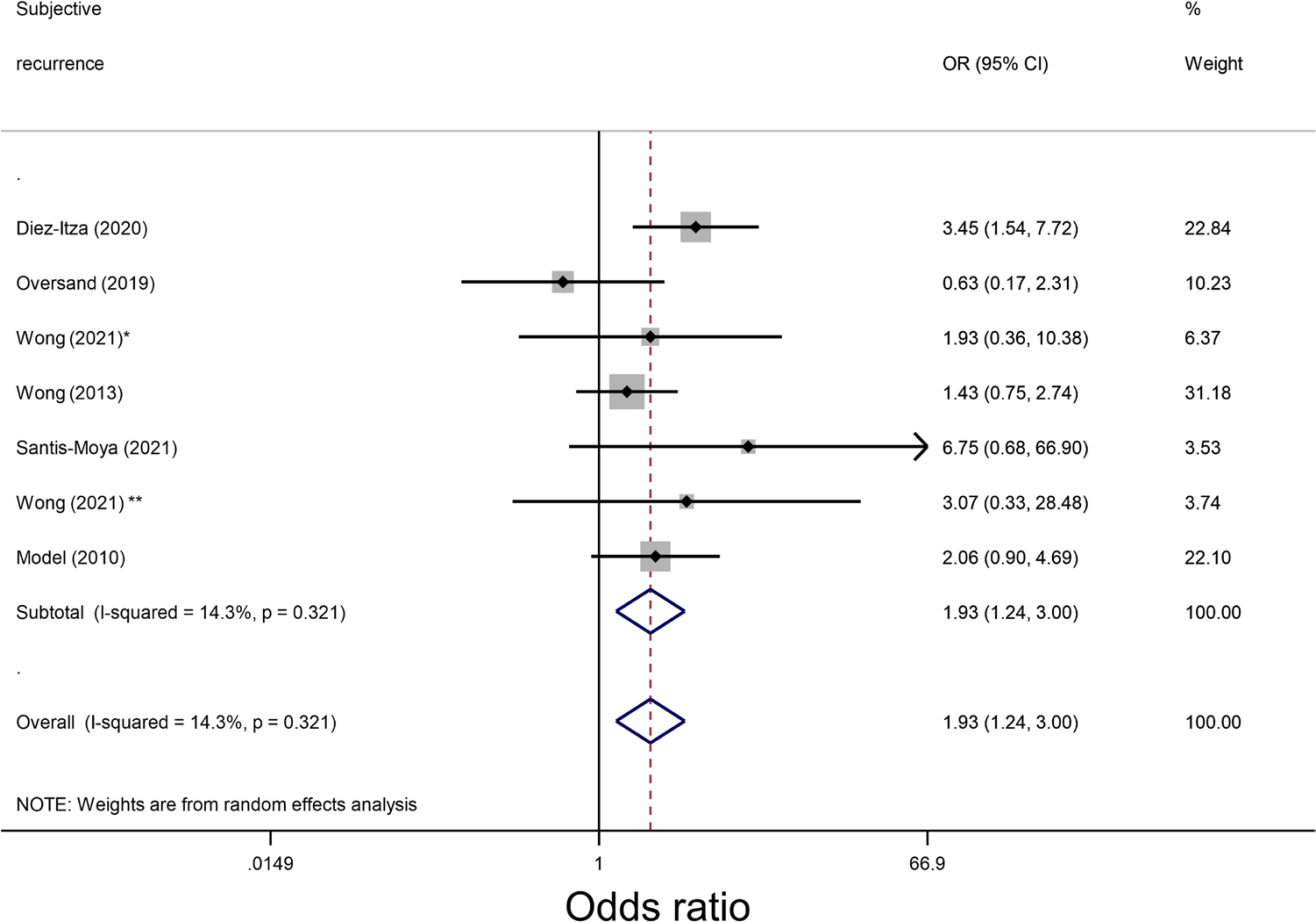
Forest plot comparing unadjusted odds ratios (ORs) for subjective prolapse recurrence in patients with levator ani muscle avulsion. Summary of unadjusted odds ratios of papers reporting subjective prolapse recurrence. *One asterisk* indicates the native tissue group, *two asterisks* indicate the mesh group (vaginal and abdominal)

A forest plot depicting the unadjusted ORs for subjective recurrence can be seen in Fig. 2. Seven studies published ORs or raw data able to be analysed for LAMA risk for subjective prolapse recurrence. However, as only one single study [31] adjusted for confounding factors, we were unable to pool adjusted odds ratios.

### Prolapse recurrence: objective any-compartment

**Fig. 3:**
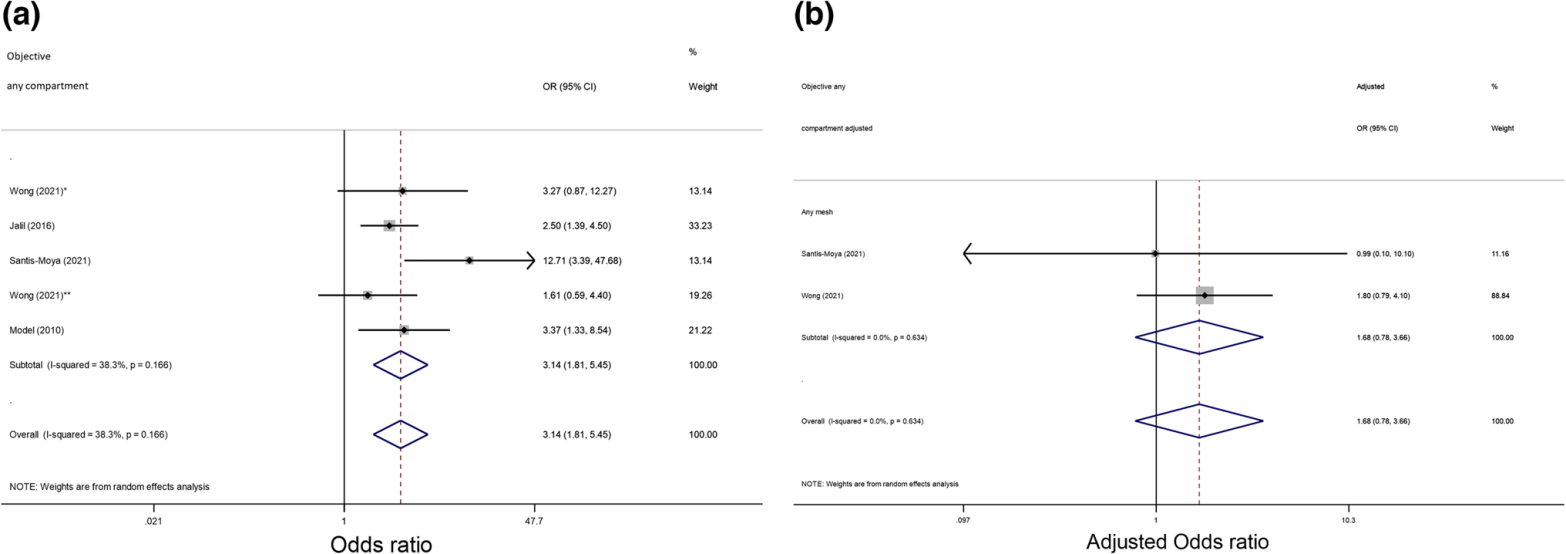
Forest plot comparing unadjusted (**a**) and adjusted (**b**) odds ratios (ORs) for objective any-compartment prolapse recurrence in patients with levator ani muscle avulsion. Summary of odds ratios (unadjusted and adjusted) of papers reporting objective any-compartment prolapse recurrence. *One asterisk* indicates the native tissue group, *two asterisks* indicate the mesh group (vaginal and abdominal)

Although univariate objective assessment demonstrated greater odds of any-compartment prolapse recurrence, after adjustment for confounding factors, the pooled adjusted analysis was no longer significant (aOR 1.68, 95% CI 0.78–3.66, 2 trials, *n* = 576, *I*^*2*^ = 0.0%, moderate quality evidence; Fig. 3).

### Reoperation

Three studies [12, 31, 33] described reoperation rates for prolapse recurrence when reporting follow-up results. The rate of reoperation was low in all three studies and was 1.5%, 0.9% and 1.05% respectively. The risk of reoperation in relation to levator ani muscle integrity was only reported in one paper [33]; thus, a meta-analysis could not be performed on a single value.

### Secondary outcomes

For the secondary outcome of analysing differences between surgical routes and types, there were no publications that reported outcomes after obliterative surgery. Only one paper described outcomes after abdominal reconstructive prolapse surgery [12] and as such a meta-analysis could not be performed.

Objective anterior compartment recurrence after multivariate analysis showed greater odds of recurrence in women with LAMA (OR 1.55 95% CI 1.23–1.96). One paper [30] in this category only had available raw data for objective recurrence on ultrasound. One paper [33] reported on a predefined optimal outcome for the anterior and mid compartments; the anterior optimal outcome was included in this secondary outcome analysis.

**Fig. 4:**
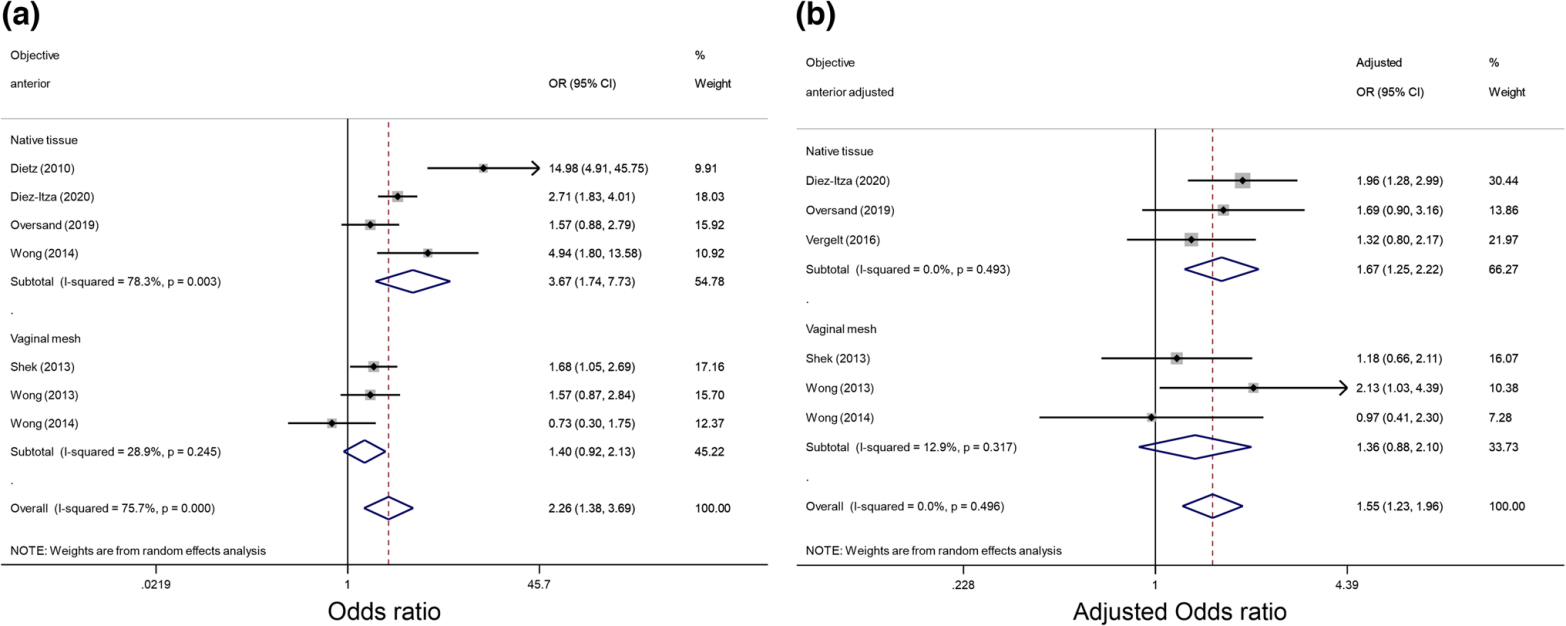
Forest plot comparing unadjusted (**a**) and adjusted (**b**) odds ratios (ORs) for objective anterior compartment prolapse recurrence after native tissue or vaginal mesh surgery in patients with levator ani muscle avulsion. Summary of odds ratios (unadjusted and adjusted) of papers in subgroup analysis reporting objective anterior compartment-only prolapse recurrence

Further subgroup analysis was conducted to determine if there was a difference in objective outcomes between native tissue and vaginal mesh surgeries for the anterior compartment as seen in Fig. 4. This showed a greater odds of an objective anterior prolapse recurrence after native tissue surgery in women with LAMA (OR 1.67, 95% CI 1.25–2.22). This same association was not demonstrated after anterior vaginal mesh surgery with a combined odds ratio not reaching statistical significance.

Adjustment for the known risk factor of a high preoperative grade of prolapse was not conducted in four of the studies in this subgroup [30, 35–37].

All studies showed moderate heterogeneity (*I*^*2*^ = 55.9%). Objective anterior compartment had the greatest heterogeneity (*I*^*2*^=75.7%), but this was due to a single smaller study by Dietz et al. [30] with an OR of 14.98, which was only observed in the pooled-unadjusted ORs.

### Risk of bias of the studies included

**Fig. 5:**
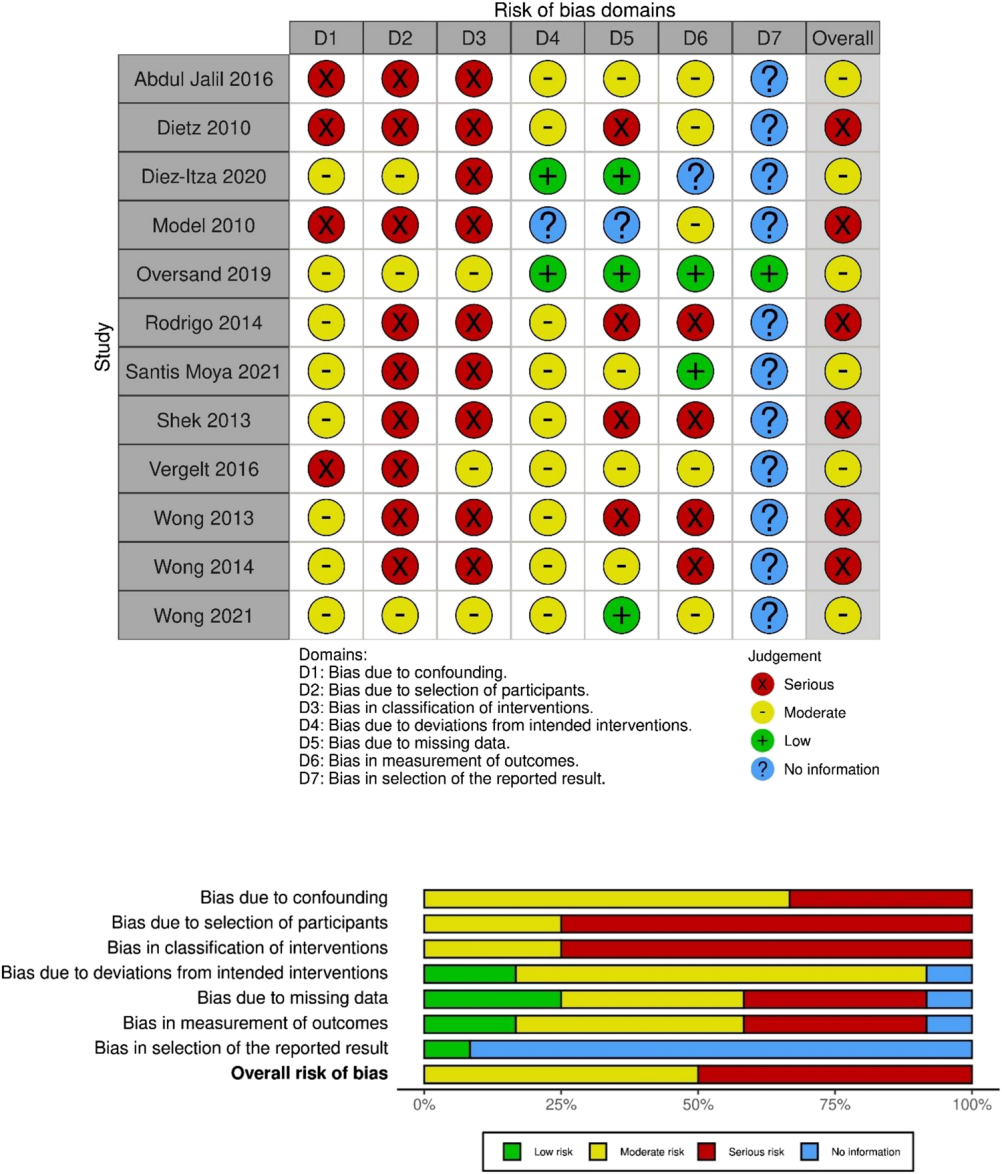
Risk of bias summary. Visual representation using the robvis visualisation tool of risk of bias for all studies included in the meta-analysis

The risk of bias of each study is summarized in Fig. 5. There were no randomized controlled studies; thus, risk of bias for confounding was at least “moderate” for all studies. Retrospective studies carry a higher risk than prospective studies for selection and classification bias so were scored as “serious” risk. Most studies adjusted for confounding factors with multivariate logistics regressions. Those that did not adjust were rated as having a “serious” risk of bias. Adjustment for confounding was not uniform in all the studies. Importantly, known risk factors for prolapse recurrence such as high preoperative stage of prolapse was adjusted for in only four studies [12, 28, 31, 33]. Specific details of factors that were adjusted for in each study are presented in Supplementary Table 2. Bias due to missing data varied greatly between studies, from nearly no loss in participants at follow-up to a nearly 70% loss in patients at follow-up assessment [37]. Only one of the prospective studies [33] had pre-registered the trial protocol prior to conducting the study.

Overall, 50% of papers included in this meta-analysis were considered high risk, whereas the other 50% inferred a moderate risk of bias.

**Fig. 6:**
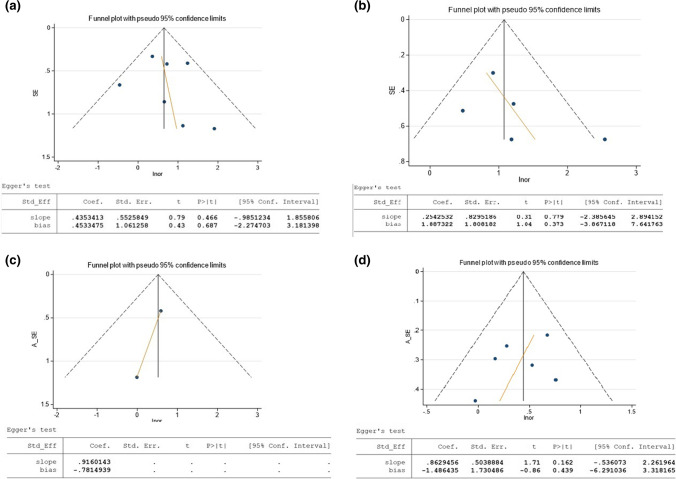
Funnel plot and Egger regression test for **a** subjective recurrence (unadjusted), **b** objective any-compartment recurrence (unadjusted), **c** objective any-compartment recurrence (adjusted) and **d** adjusted objective anterior compartment-only recurrence. Summary of publication bias presented as funnel plots and Egger regression for subjective recurrence, objective any-compartment recurrence (unadjusted and adjusted) and objective anterior compartment-only recurrence

A funnel plot was created to examine publication bias in the outcomes of risk of subjective recurrence, objective any-compartment recurrence (unadjusted and adjusted) and objective anterior compartment recurrence (adjusted). The funnel plot appeared slightly asymmetric, with smaller studies tending to have larger ORs (Fig. 6). The estimated Egger’s regression bias coefficient is 0.886 (SE=0.813), and a *p* value of 0.291. This suggests no evidence of the presence of a small-study effect.

### Certainty of evidence

**Fig. 7:**
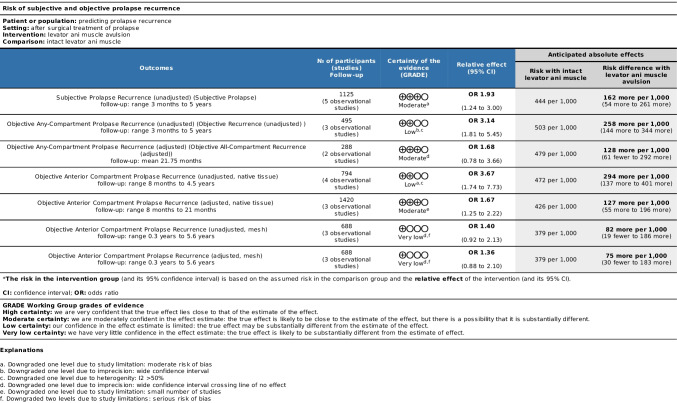
Summary of findings

In general, the evidence was moderate to low quality with most downgraded owing to bias and imprecision or a small number of studies contributing to the outcome. These findings are summarised in Fig. 7. One paper could not be included in this figure [32] as the incidence of levator muscle avulsion was not reported in the study.

## Discussion

This systematic review and meta-analysis evaluated 12 studies, of which 9 were retrospective and 3 were prospective. There was a total of 2,637 subjects with a follow-up period between 0.3 and 6.4 years. The studies included in this meta-analysis showed moderate heterogeneity and were of moderate to low quality owing to bias, as shown in Fig. 7.

### Principal findings and comparison with existing literature

After pooled adjusted analysis, LAMA was not a risk factor for objective any-compartment recurrence. There were insufficient data to draw a conclusion in relation to the risk of subjective recurrence or reoperation.

On subgroup analysis, LAMA was shown to increase the risk of anterior compartment prolapse recurrence after native tissue repair, but not after transvaginal mesh.

Our findings are not consistent with the most recent systematic review [5]. Older reviews have not been able to draw a conclusion owing to a lack of data [4]. This inconsistency may be able to be explained by methodological differences between the systematic reviews. Our review specifically sought out adjusted results to ensure that known confounding factors are accounted for and used pre-defined outcomes that are meaningful to both clinicians and patients, as endorsed by IUGA/ICS and Cochrane reviews in the surgical management of prolapse. Our review also includes more recent studies, which have shown less association between LAMA and prolapse recurrence.

Higher preoperative prolapse stage, family history and previous prolapse repairs have all been demonstrated to increase the likelihood of prolapse recurrence [4–6], yet many studies did not adjust for these factors in their analyses. In the studies included, only 4 studies [12, 28, 31, 33] adjusted for high grade of prolapse and 1 [38] study recruited only patients with stage 3–4 prolapse. No studies adjusted for family history. Of the 5 studies [12, 30, 34, 36, 37] that included repeat prolapse repairs as part of their cohort, none adjusted for this factor in their logistics regression. In addition, papers performed adjusted regressions only if a statistically significant value was found on univariate regressions. This limited the number of values available to combine for a pooled adjusted meta-analysis. The variation in the factors used for adjustment of confounding factors and the lack of uniformity between studies means that although adjusted ORs have been presented, these may not necessarily be a true reflection of the real risk of LAMA in prolapse recurrence. Yet, if we were to consider only the five studies that adjusted for one recurrent prolapse risk factor of (high preoperative grade of prolapse), it can be noted that there would be no change in results for the findings in the categories of adjusted any-compartment recurrence (Fig. 3b) and the adjusted objective anterior compartment recurrence after native tissue repair (Fig. 4b).

In a clinical setting, recurrent prolapse that is significant and bothersome to a patient would present with symptoms correlating with subjective recurrence or reoperation. The finding of an objective recurrence on examination or ultrasound without symptoms may not necessitate further treatment and hence be, arguably, a less clinically meaningful finding. Reporting of reoperation rates and subjective recurrence rates may be a more pragmatic way of defining whether LAMA is a risk factor for prolapse recurrence. Unfortunately, literature was scarce and there was insufficient information to perform a meta-analysis of the adjusted ORs for subjective recurrence in our study. Reoperation rates were not reported for women with and without LAMA in any of the papers included in this review and as such a meta-analysis could not be performed.

Objective recurrence in eight studies in this review was defined as that occurring only in the anterior compartment. Single-site repairs have previously been shown to increase the risk of prolapse in other compartments [40, 41]. Hence, this could represent an underestimation of true post-surgical objective outcomes.

The quality of the studies included varied significantly in this review. The level of evidence was low, with most studies being retrospective in nature. In addition, there was a significant number of patients who were lost to follow-up in the retrospective trials, thus increasing the risk of bias. Overall, the studies were at moderate or severe risk of bias.

### Strengths and limitations

The strength of this study lies in the new information that it presents differing from previous published papers and meta-analyses [4, 5]. Our study presents a more in-depth investigation into the risk of prolapse recurrence in women with LAMA through pre-defined clinically significant outcomes and detailed subgroup analysis. We have combined adjusted ORs rather than unadjusted ORs alone, thus reducing the risk of bias owing to confounding. Our study also included the most recent trials [12, 31, 33, 38] that have been published since the last systematic review and meta-analysis [5].

The study was limited by the availability of data to allow analysis of levator integrity in each cohort of women in the articles that were included. Furthermore, the absence of high-quality data and weaker retrospective study designs place the conclusions at risk of significant bias and need to be considered when interpreting the results from this study. The large variation in surgical techniques with the majority of those being reconstructive vaginal prolapse repair methods means that extrapolating this study to laparoscopic techniques, Manchester repairs, or even obliterative techniques (which have not been studied), needs to be done with caution. Meaningful conclusions that are relevant to modern prolapse repair techniques or non-reconstructive vaginal prolapse procedures are difficult with the limited evidence available.

## Conclusions

The risk of subjective prolapse recurrence, objective prolapse recurrence and reoperation in women who have LAMA remains unclear after pooled analysis of adjusted ORs. Subgroup analysis of objective anterior compartment prolapse recurrence appears to be increased with levator avulsion injuries after native tissue repairs. Further prospective or randomised studies are warranted to further explore recurrence risks in women with LAMA after abdominal or laparoscopic prolapse repairs or non-vaginal reconstructive techniques. In addition, when building a methodology, consideration should be given to analysing important potential confounding factors and investigating prolapse recurrence defined by subjective recurrence, objective recurrence and reoperation rates.

## Supplementary information

ESM 1 (DOCX 15 kb)

ESM 2 (DOCX 17 kb)
