# Supplementary material for: Is levator ani avulsion a risk factor for prolapse recurrence? A systematic review and meta-analysis
Source: Int Urogynecol J. 2022 May 10;33(7):1813–26. doi: 10.1007/s00192-022-05217-2 (PMC9270296; doi:10.1007/s00192-022-05217-2)
Supplement: Supplementary file 1 — (DOCX 15 kb) [file 192_2022_5217_MOESM1_ESM.docx]

**Supplementary material 1:** Search strategies for all databases

Searches conducted 18 April 2021

| **PubMed** | **Embase** | **Cochrane** |
| --- | --- | --- |
| ("risk factors"[MeSH Terms] OR ("risk"[All Fields] AND "factors"[All Fields]) OR "risk factors"[All Fields]) AND (("prolapse"[MeSH Terms] OR "prolapse"[All Fields] OR "prolapses"[All Fields] OR "prolapsed"[All Fields] OR "prolapsing"[All Fields]) AND ("recurrance"[All Fields] OR "recurrence"[MeSH Terms] OR "recurrence"[All Fields] OR "recurrences"[All Fields] OR "recurrencies"[All Fields] OR "recurrency"[All Fields] OR "recurrent"[All Fields] OR "recurrently"[All Fields] OR "recurrents"[All Fields])) AND ((("levator"[All Fields] OR "levators"[All Fields]) AND "ani"[All Fields]) OR "puborectalis"[All Fields])  **Results:** 24  ("levator"[All Fields] OR "levators"[All Fields]) AND (("prolapse"[MeSH Terms] OR "prolapse"[All Fields] OR "prolapses"[All Fields] OR "prolapsed"[All Fields] OR "prolapsing"[All Fields]) AND ("recurrance"[All Fields] OR "recurrence"[MeSH Terms] OR "recurrence"[All Fields] OR "recurrences"[All Fields] OR "recurrencies"[All Fields] OR "recurrency"[All Fields] OR "recurrent"[All Fields] OR "recurrently"[All Fields] OR "recurrents"[All Fields])) AND ("surgery"[MeSH Subheading] OR "surgery"[All Fields] OR "surgical procedures, operative"[MeSH Terms] OR ("surgical"[All Fields] AND "procedures"[All Fields] AND "operative"[All Fields])  **Results:** 114  ("levator"[All Fields] OR "levators"[All Fields]) AND ("avulse"[All Fields] OR "avulsed"[All Fields] OR "avulsing"[All Fields] OR "avulsive"[All Fields] OR "fractures, avulsion"[MeSH Terms] OR ("fractures"[All Fields] AND "avulsion"[All Fields]) OR "avulsion fractures"[All Fields] OR "avulsion"[All Fields] OR "avulsions"[All Fields]) AND ("surgery"[MeSH Subheading] OR "surgery"[All Fields] OR "surgical procedures, operative"[MeSH Terms] OR ("surgical"[All Fields] AND "procedures"[All Fields] AND "operative"[All Fields]) OR "operative surgical procedures"[All Fields] OR "general surgery"[MeSH Terms])  **Results:** 146  Combined searches: 244 results | (“levator avulsion” [All fields] AND surgery [All fields])  **Results:** 113 articles  (“levator” [All fields] AND “prolapse” [All fields] AND “recurrence” [All fields] AND “surgery” [All fields])  **Results:** 217 articles  (risk AND factors AND prolapse AND recurrence AND (levator AND ani OR puborectalis)  **Results:** 34  Combined above 3 searches  **Results:** 291 studies | MeSH descriptor: [Pelvic Organ Prolapse] explode all trees  **Results:** 11 articles  (“Pelvic organ prolapse” [All fields] AND “ levator”)  **Results:** 3 articles  (pelvic organ prolapse):kw AND (recurrence):kw  **Results:** 2 articles |
