# Supplementary material for: Is levator ani avulsion a risk factor for prolapse recurrence? A systematic review and meta-analysis
Source: Int Urogynecol J. 2022 May 10;33(7):1813–26. doi: 10.1007/s00192-022-05217-2 (PMC9270296; doi:10.1007/s00192-022-05217-2)
Supplement: Supplementary file 2 — (DOCX 17 kb) [file 192_2022_5217_MOESM2_ESM.docx]

**Supplementary Table 2: Variables included in adjusted analyses**

|  | Age | Menopause Status | Stage of Prolapse (3/4) | LAMA | Hiatal area/Ballooning | BMI | Length of follow-up | Family History | Previous vaginal surgery | Mesh Type/Use | Other variables * |
| --- | --- | --- | --- | --- | --- | --- | --- | --- | --- | --- | --- |
| Abdul Jalil 2016 (29) | - | - | - | - | - | - | - | - | - | - | - |
| Dietz 2010 (30) | - | - | - | - | - | - | - | - | - | - | - |
| Diez-Itza 2020 (31) | + | + | + | + | + | - | - | - | - | - | - |
|  |  |  |  |  |  |  |  |  |  |  |  |
| Model 2010 (32) | - | - | - | - | - | - | - | - | - | - | - |
| Oversand 2019 (33) | + | - | + | + | - | - | - | - | - | - | + |
| Rodrigo 2014 (34) | - | - | - | + | + | - | - | - | - | + | - |
| Santis Moya 2021 (12) | + | - | + | + | + | + | - | - | - | - | + |
| Shek 2013 (35) | - | - | - | + | + | - | + | - | - | + | - |
| Vergeldt 2016 (28) | - | - | + | + | + | - | - | - | - | - | + |
| Wong 2013 (36) | + | - | - | + | + | + | - | - | + | - | + |
| Wong 2014 (37) | + | - | - | + | + | + | - | - | + | - | + |
| Wong 2021 (38) | - | - | ^ | + | + | - | + | - | - | + | - |

* Other variables:
Oversand: local oestrogen, chronic disease, PFDI20 score
Santis-Moya: instrumental delivery
Vergeldt: vaginal assisted delivery, number of compartments
Wong 2013, Wong 2014: parity

^note: patient cohort only consisted of stage 3-4 prolapse
